# Prone position in the mechanical ventilation of acute respiratory distress syndrome children: a systematic review and meta-analysis

**DOI:** 10.3389/fped.2024.1293453

**Published:** 2024-03-07

**Authors:** Wen Qin, Lei Mao, Yue Shen, Li Zhao

**Affiliations:** ^1^ Department of Emergency, Children’s Hospital of Nanjing Medical University, Nanjing, Jiangsu, China; ^2^ PICU, Children’s Hospital of Nanjing Medical University, Nanjing, Jiangsu, China

**Keywords:** prone position, mechanical ventilation, children, acute respiratory distress syndrome, treatment, care, nursing

## Abstract

**Background:**

Prone position has been well recognized for the treatment of adult acute respiratory distress syndrome (ARDS). We aimed to evaluate the role of prone position in the mechanical ventilation in children with ARDS, to provide evidence to the treatment and care of children with ARDS.

**Methods:**

We searched the Pubmed et al. databases by computer until January 23, 2024 for randomized controlled trials (RCTs) on the role of prone position in the mechanical ventilation in children with ARDS. We evaluated the quality of included studies according to the quality evaluation criteria recommended by the Cochrane library. RevMan 5.3 software was used for meta-analysis.

**Results:**

7 RCTs involving 433 children with ARDS were included. Meta-analysis indicated that prone position is beneficial to improve the arterial oxygenation pressure [MD = 4.27 mmHg, 95% CI (3.49, 5.06)], PaO_2_/FiO_2_ [MD = 26.97, 95% CI (19.17, 34.77)], reduced the oxygenation index [MD = −3.52, 95% CI (−5.41, −1.64)], mean airway pressure [MD = −1.91 cmH_2_O, 95% CI (−2.27, −1.55)] and mortality [OR = 0.33, 95% CI (0.15, 0.73), all *P* < 0.05]. There were no statistical differences in the duration of mechanical ventilation between the prone position group and control group [MD = −17.01, 97.27, 95% CI (−38.28, 4.26), *P* = 0.12]. Egger test results showed that no significant publication bias was found (all *P* > 0.05).

**Conclusions:**

Prone position ventilation has obvious advantages in improving oxygenation, but there is no significant improvement in the time of mechanical ventilation in the treatment of children with ARDS. In the future, more large-sample, high-quality RCTs are still needed to further analyze the role of prone position in the mechanical ventilation in children with ARDS.

## Introduction

Acute respiratory distress syndrome (ARDS) is an acute diffuse alveolar lesion caused by a variety of direct or indirect factors with acute respiratory failure as the main clinical manifestation (1). The main pathological manifestations were injury of alveolar epithelial cells and capillary endothelial cells, increase of intercellular permeability and infiltration of inflammatory cells (2). This kind of children are the main patients in pediatric intensive care unit (PICU), and the mortality rate is high. The prevalence rate of pediatric ARDS ranges from 0.7% to 4.5%, and the case fatality rate can up to 60.3% (3, 4). Although there are similar pathophysiological changes between children and adult patients, there are great differences in the incidence, risk factors, mechanical ventilation mode setting and prognosis. The treatment guidelines for adult ARDS suggest that adding other auxiliary ventilation modes to the traditional supine mechanical ventilation mode can help to improve the prognosis of ARDS patients, including high frequency oscillatory ventilation, prone position ventilation, lung expansion, extracorporeal membrane lung and so on (5). In particular, it is suggested that early prone position ventilation in patients with severe ARDS may improve pulmonary ventilatory blood flow ratio, promote sputum drainage and reduce mortality (6). However, there is no definite evidence that prone position ventilation can improve the prognosis of children with ARDS (7).

Prone position ventilation was put forward by Bryan in 1974. Prone position is adopted during mechanical ventilation to improve dorsal lung tissue ventilation and to make whole lung ventilation more uniform. Prone position ventilation, as an important lung protective ventilation strategy, has been widely used in patients with adult respiratory failure. The results showed that arterial oxygen saturation, partial pressure of oxygen and oxygenation index were significantly improved after changing posture (8). In recent years, there are different studies on prone position ventilation in the treatment of all kinds of respiratory failure patients with mechanical ventilation. In general, most studies have confirmed the positive role of prone position ventilation in mechanical ventilation in patients with respiratory failure (9). Recent systematic review (10) has included six randomized controlled trials (RCTs) and come to the finding that although the included RCTs suggest that prone positioning may offer some advantage, there is little evidence to make definitive recommendations. So far, there is still much controversy about whether children with ARDS should routinely use prone position ventilation. However, prone position ventilation has been routinely carried out in adult patients, and has achieved good results. Children have lighter body weight and better cardiopulmonary compensatory function, it's necessary to understand that whether prone position ventilation should be routinely performed in children with ARDS (11). Therefore, this study aimed to conduct meta-analysis to evaluate the clinical therapeutic effect of prone position ventilation in children with ARDS, to guide the treatment and nursing care of children with ARDS.

## Methods

This study was conducted and reported in comply with the Preferred Reporting Items for Systematic reviews and Meta-Analyses (PRISMA) statement (12).

### Literature inclusion and exclusion criteria

The inclusion criteria of this meta-analysis were as follows: the type of study was a published RCT of mechanical ventilation in prone position in children with acute respiratory distress syndrome (ARDS). The study population were children aged 0–18 years, who met the general diagnostic criteria of ARDS: onset or new respiratory symptoms with PaO_2_/FiO_2_ <300 mmHg (13, 14). In all studies, invasive mechanical ventilation and prone position were performed within 48 h after the diagnosis of ARDS, and prone position ventilation was performed for at least 4 h a day. The RCT reported the related outcome indicators including the results of blood gas analysis (oxygen partial pressure, carbon dioxide partial pressure) and ventilator parameters (mean airway pressure, oxygen concentration, positive end-expiratory pressure, tidal volume) and the duration of mechanical ventilation and mortality.

We excluded the following related literatures: literatures in the form of abstracts or reports that were not published in full text; clinical controlled trials of patients in the prone position before and after ventilation; studies with incomplete data or we positively contacted with the author to ask for the original data without results; adult studies.

### Literature search

The two researchers (Wen Qin and Lei Mao) independently searched the Pubmed, Clinical trials, EMBASE, Cochrane Library, Medine, Ovid Chinese Biomedical Literature Database, Wanfang, Weipu and China knowledge Network by computer until January 23, 2024. The search strategies used in this meta-analysis were: (“prone position” OR “proning”) AND (“ARDS” OR “acute respiratory distress syndrome” OR “acute lung injury”) AND (“children” OR “child” OR “pediatric”). This meta-analysis used the combination of free words, MeSH subject words and Boolean logic operators to establish the relevant retrieval formula of each database. At the same time, the researchers (Wen Qin and Lei Mao) conducted manual search to comprehensively search the relevant literatures in the included RCTs and important reviews.

### Data extraction

This study used Endnote document manager to manage the final literature included in the study. If the literature information was incomplete, we would contact the author for information. Two researchers (Wen Qin and Lei Mao) carried out data extraction and literature evaluation on the included RCTs respectively. The data extracted by this meta-analysis included the first author, the year of publication, the age of the child, the details of the intervention and related outcome indicators.

### Quality evaluation

Two researchers (Wen Qin and Lei Mao) independently evaluated all selected studies according to the quality evaluation criteria recommended by the Cochrane library. Repeated literature was excluded in the initial examination, and all potential related studies were reviewed and analyzed later, mainly aimed at random methods, allocation hiding, blind method, result data, selective publication. The results were expressed as low, unclear and high. If there are differences in the evaluation results of the two researchers, an agreement would be reached through discussion. And if no consensus was reached, the third researcher would be asked for arbitration. For those with incomplete information report, we tried to contact the corresponding author to supplement the relevant information.

### Statistical analysis

RevMan 5.3.0 software provided by Cochrane collaboration network was used for meta-analysis. The continuous variables were expressed by mean difference (MD), and mortality was expressed by odds ratio (OR). Chi-square test was used to judge heterogeneity, and statistical heterogeneity was judged according to *I*^2^ value and *P*-value. If the heterogeneity was small (*I*^2 ^< 50%, *P* > 0.01), fixed effect model was used for data combination analysis. If there was statistical heterogeneity (*I*^2 ^≥ 50%, *P* < 0.01), random effect model was used. Funnel plot and Egger tests were performed to detect the publication bias. There was significant statistical difference between the two groups when *P* < 0.05.

## Results

### RCT inclusion

**Figure 1:**
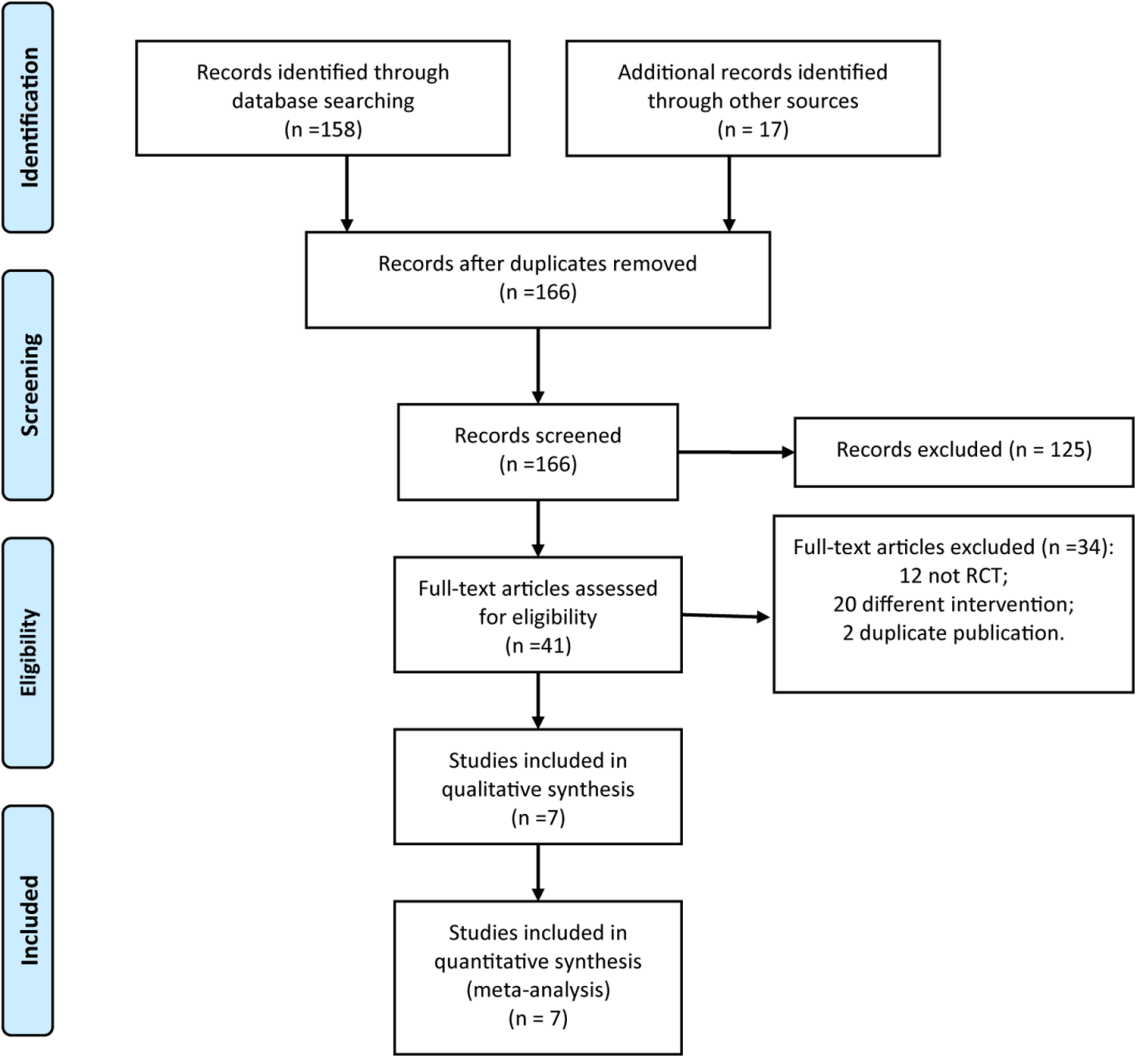
PRISMA flow diagram of study selection.

The two researchers (Wen Qin and Lei Mao) searched a total of 175 relevant research reports for the first time according to the relevant key words. They had contacted the corresponding authors of four papers for more details. After reading the title and abstract of the article, they excluded the irrelevant literatures, and then screened it by reading the abstract and the full text, and we finally included 7 RCTs (15–21). The literature screening process is shown in Figure 1.

### The characteristics of included RCTs

As shown in Table 1, a total of 433 children with ARDS were included in the 7 studies (15–21). 217 children received prone position ventilation and 216 children received supine position ventilation. All the studies were carried out in the central ICU of the children's hospital. The duration of prone position ventilation was different in each study, but the duration of prone position ventilation was more than 4 h per day.

**Table 1:** The characteristics of included studies.

Study	Sample size		Age	ARDS diagnosis (PaO_2_/FiO_2_, mmHg)	Interventions		Time points that the outcomes were measured and compared
	Prone position group	Control group			Prone position group	Control group	
Curley et al., 2005	51	51	2–18 years	<200	Prone position >20 h/days	Supine position	1, 2 days after intervention
Dong et al., 2015	33	32	2–10 years	<200	Prone position >10 h/days	Supine position	1, 2, 3,4 days after intervention
Ibrahim and Elmohamady, 2007	11	11	8–10 years	<200	Prone position for 20 h/days	Supine position	1, 2 days after intervention
Liu and Zhang, 2018	31	31	1–2 years	<200	Prone position >4 h/days	Supine position	1, 2, 3, 7 days after intervention
Sawhney et al., 2005	22	21	0–12 years	<200	Prone position >4 h/days	Supine position	3 days after intervention
Sun et al., 2017	36	36	2–10 years	<300	Prone position >6 h/days	Supine position	1, 2, 7 days after intervention
Wu et al., 2015	33	34	0–21 days	<200	Prone position for 20 h/days	Supine position	1, 2, 3, 5 days after intervention

### The quality of included RCTs

**Figure 2:**
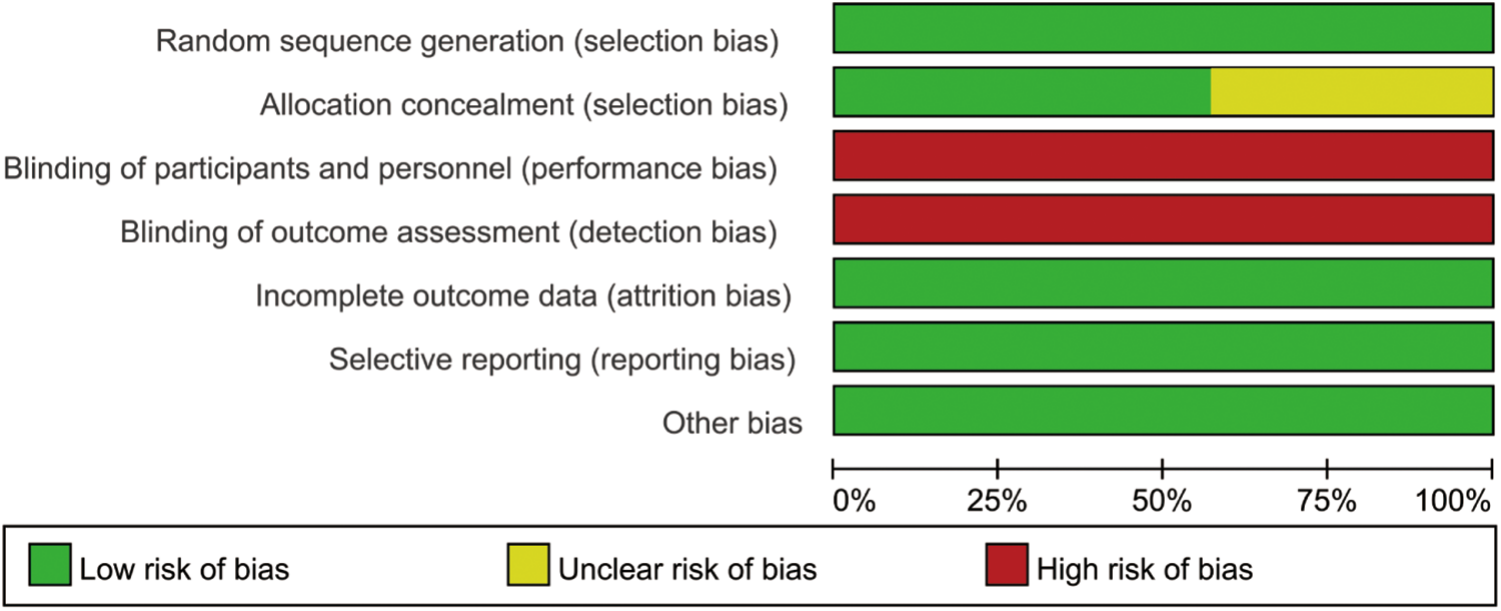
Risk of bias graph.

**Figure 3:**
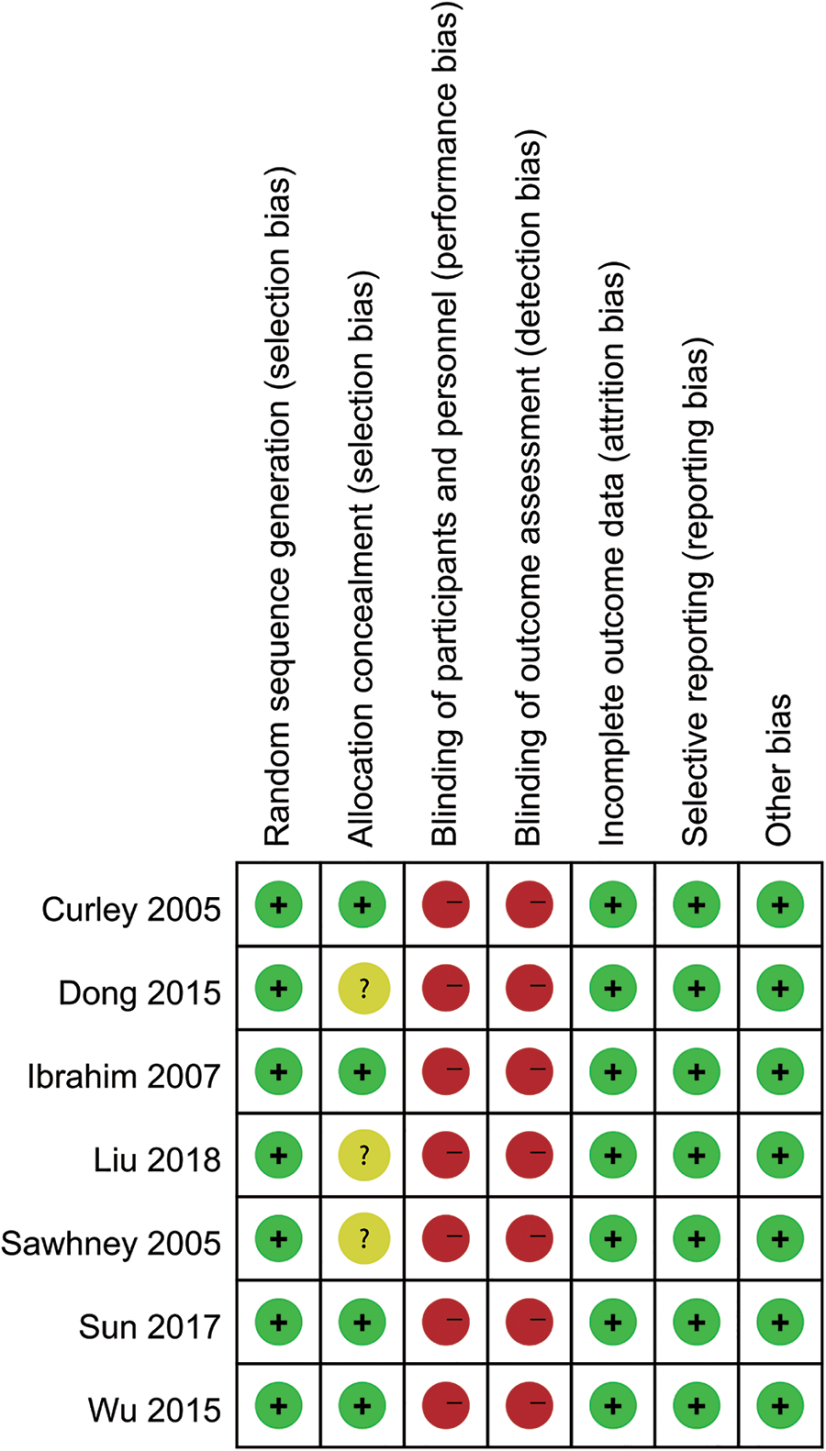
Risk of bias summary.

The quality evaluation results of the articles included in RCTs are shown in Figures 2, 3. Although all studies mentioned randomized controlled grouping, 3 studies did not mention specific allocation hiding methods. Due to the particularity of prone position ventilation intervention, all studies did not implement blind method. No other biases were found among the included RCTs.

### Meta-analysis

**Figure 4:**
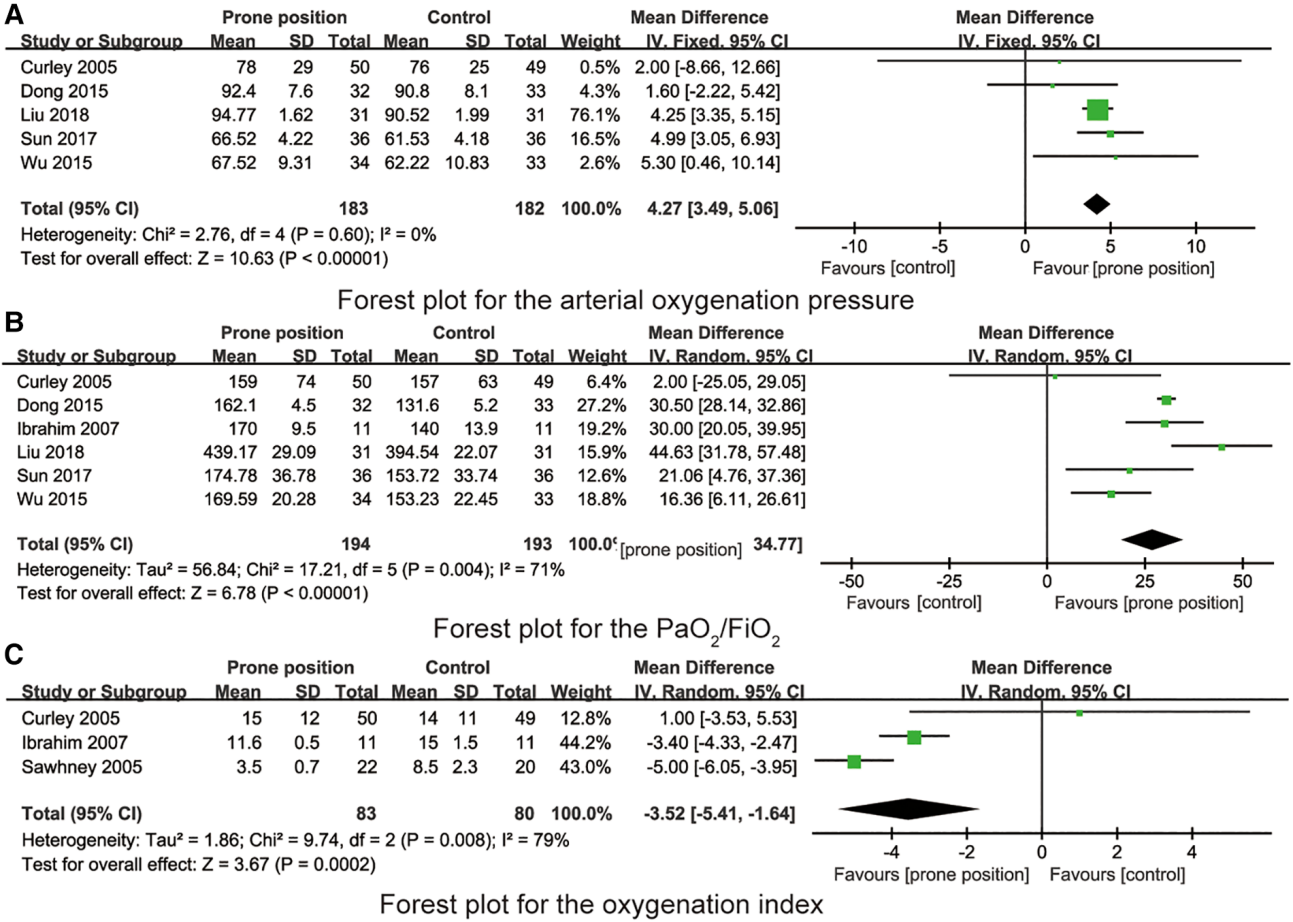
(**A**) Forest plot for the arterial oxygenation pressure. (**B**) Forest plot for the PaO_2_/FiO_2_. (**C**) Forest plot for the oxygenation index.

5 studies reported the effect of prone position ventilation on arterial oxygenation pressure in children with ARDS. There was no statistical heterogeneity among the studies. Using the fixed effect model, the arterial oxygenation pressure of children with prone position ventilation was significantly higher than that in the control group [MD = 4.27 mmHg, 95% CI (3.49, 5.06), *P* < 0.001, Figure 4A].

6 studies reported the effect of prone position ventilation on PaO_2_/FiO_2_ in children with ARDS. There was statistical heterogeneity among the studies. Using the random effect model, the PaO_2_/FiO_2_ of children with prone position ventilation was significantly higher than that in the control group [MD = 26.97, 95% CI (19.17, 34.77), *P* = 0.004, Figure 4B].

3 studies reported the effect of prone position ventilation on oxygenation index in children with ARDS. There was statistical heterogeneity among the studies. Using the random effect model, the oxygenation index of children with prone position ventilation was significantly lower than that in the control group [MD = −3.52, 95% CI (−5.41, −1.64), *P* = 0.008, Figure 4C].

**Figure 5:**
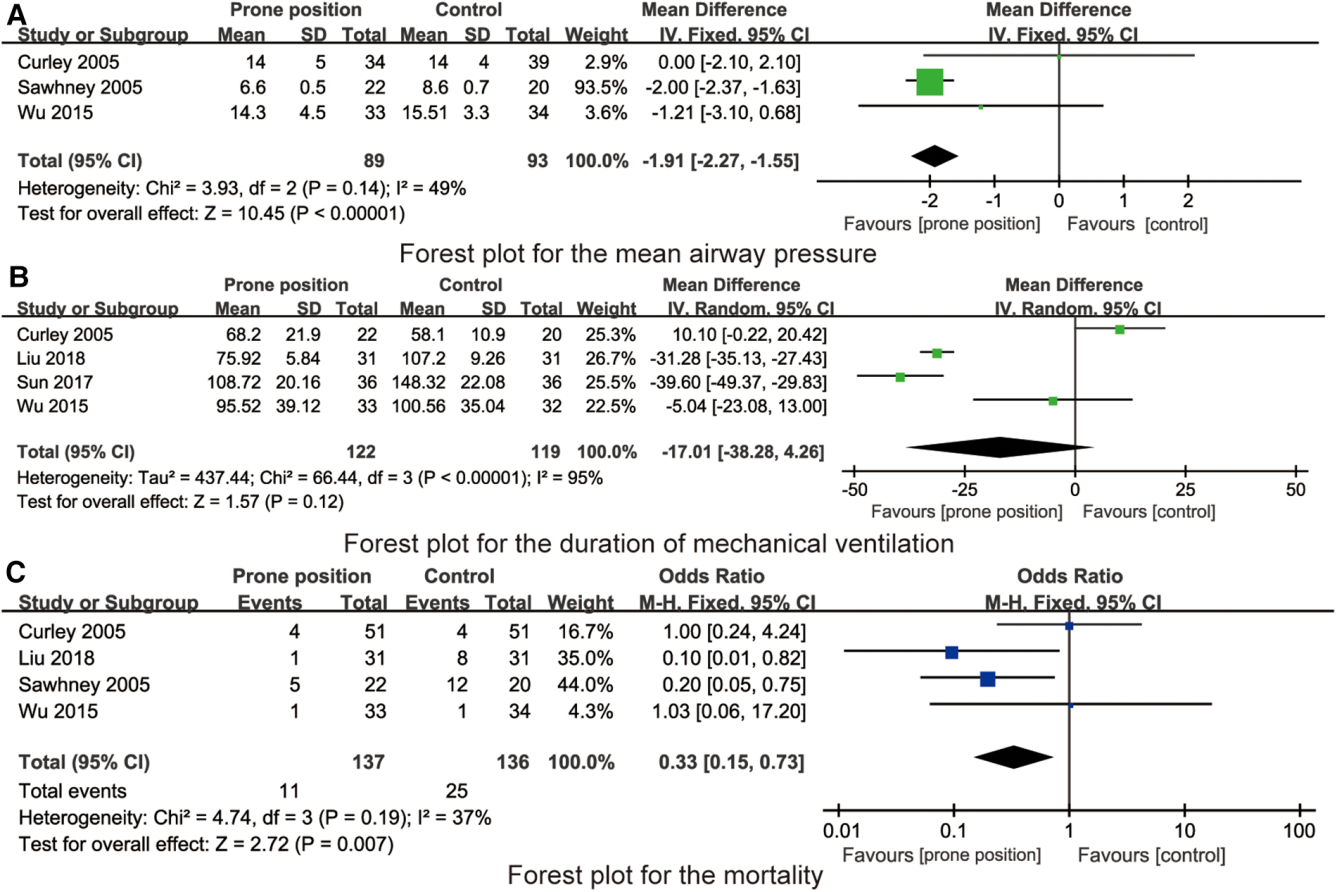
(**A**) Forest plot for the mean airway pressure. (**B**) Forest plot for the duration of mechanical ventilation. (**C**) Forest plot for the mortality.

3 studies reported the effect of prone position ventilation on mean airway pressure in children with ARDS. There was no statistical heterogeneity among the studies. Using the fixed effect model, the mean airway pressure of children with prone position ventilation was significantly lower than that in the control group [MD = −1.91 cmH_2_O, 95% CI (−2.27, −1.55), *P* < 0.001, Figure 5A].

4 studies reported the effect of prone position ventilation on PaO_2_/FiO_2_ in children with ARDS. There was statistical heterogeneity among the studies. Using the random effect model, there were no statistical differences in the duration of mechanical ventilation between the prone position group and control group [MD = −17.01 h, 95% CI (−38.28, 4.26), *P* = 0.12, Figure 5B].

4 studies reported the effect of prone position ventilation on mortality in children with ARDS. There was no statistical heterogeneity among the studies. Using the fixed effect model, the mortality of children with prone position ventilation was significantly lower than that in the control group [OR = 0.33, 95% CI (0.15, 0.73), *P* = 0.007, Figure 5C].

**Figure 6:**
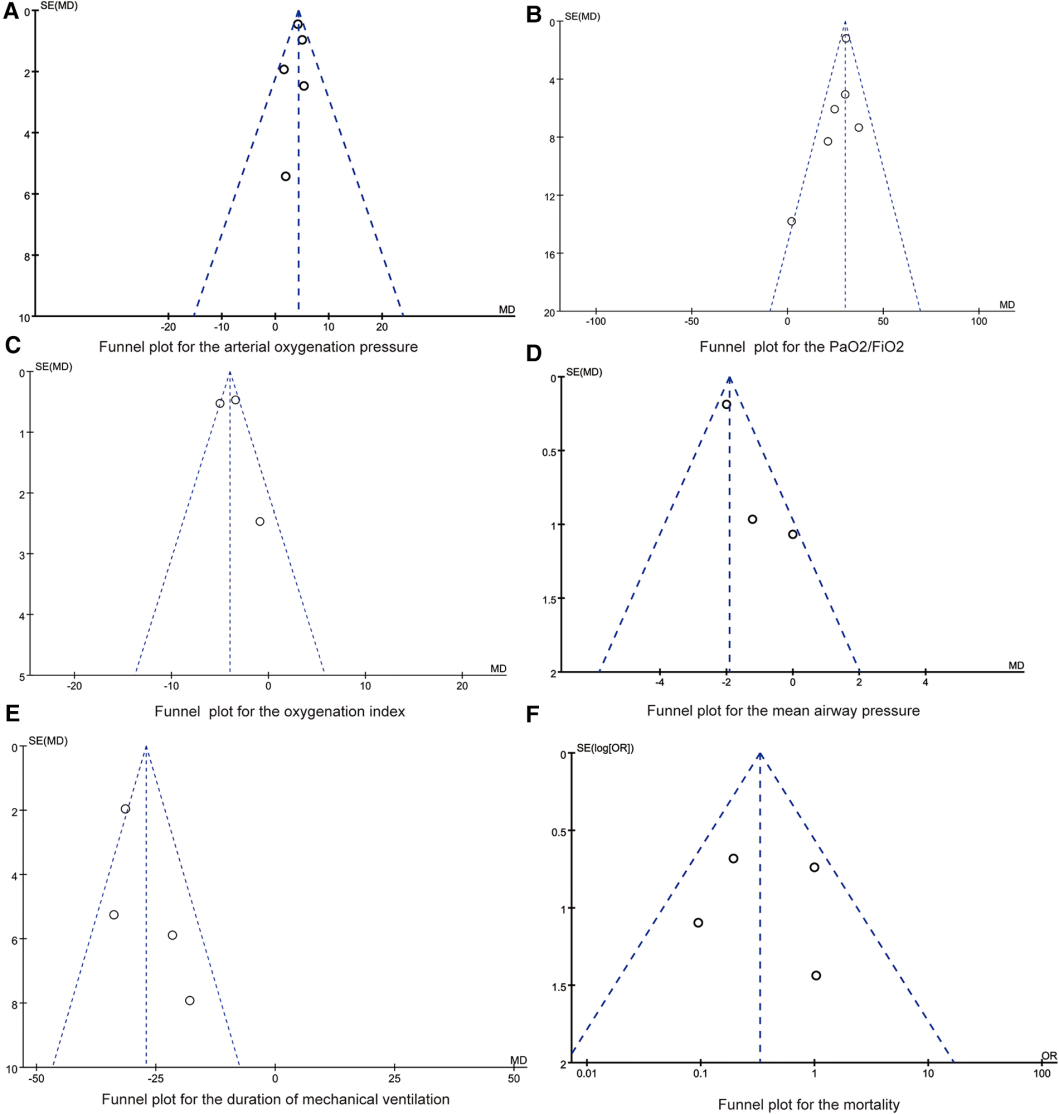
(**A**) Funnel plot for the arterial oxygenation pressure. (**B**) Funnel plot for the PaO_2_/FiO_2_. (**C**) Funnel plot for the oxygenation index. (**D**) Funnel plot for the mean airway pressure. (**E**) Funnel plot for the duration of mechanical ventilation. (**F**) Funnel plot for the mortality.

We use inverted funnel chart analysis of each outcome to evaluate the publication bias among the studies (Figure 6). The results showed that the dots in the funnel chart were basically symmetrical, indicating that the possibility of publication bias was small. Egger test results showed that no significant publication bias was found (all *P* > 0.05).

## Discussions

Prone position ventilation as an adjuvant therapy for patients with ARDS, the mechanism of improving oxygenation may be that when the patient is in prone position, the distribution of ventilated alveoli and pulmonary blood flow distribution changes, which is beneficial to improve the ventilation/blood flow ratio of patients, thus improving oxygenation (10, 22). In this study, Meta-analysis was used to evaluate the effect of prone position ventilation on physiological indexes of children with ARDS. The results showed that prone position ventilation could significantly improve the arterial oxygenation pressure, PaO_2_/FiO_2_ and oxygenation index, mean airway pressure and duration of mechanical ventilation and mortality, of children with ARDS, but had little effect on the time of mechanical ventilation in children with ARDS. Prone position ventilation can significantly improve the prognosis of children with ARDS.

Although there is no formal definition of pediatric ARDS, it is clinically considered to be the manifestation of one or more of the following signs or symptoms: shortness of breath, wheezing, accelerated respiratory rate, rapid heartbeat, chest wall retraction, chest and abdominal asynchrony (23). Respiratory distress can lead to hypoxemia, decreased partial pressure of arterial oxygen, increased partial pressure of carbon dioxide, changes in neurological state, and eventually lead to respiratory or multiple organ failure (or both), leading to death (24). The main pathological changes of ARDS were alveolar and alveolar interstitial edema, in which there were great differences in alveolar pathological changes in different regions (25, 26). The main manifestations are alveolar collapse and atelectasis in gravity-dependent areas and collapse of small airways in gravity-dependent areas, but alveolar hyperventilation in non-gravity areas (27–29). During prone ventilation, the negative pressure in the thoracic cavity gradually decreased from the dorsal to the ventral, while the transpulmonary pressure decreased, resulting in a decrease in ventral ventilation, but it could still maintain the opening of ventral alveoli (30). At the same time, after the prone position, the lung lobe volume slightly increased compared with the supine position, the original anatomical position is located below the heart compressed by the heart to reduce the lung lobe volume increased, thus causing the collapse alveoli originally oppressed by the heart to re-expand (31). Through the comparison of lung tissue ventilation blood flow gradient between healthy volunteers and mechanically ventilated patients in different positions, it has been confirmed that the ventilation distribution increase slowly from ventral lung tissue to back when lying on the back, and suddenly decreased at the last 25% of the dorsal lung tissue. The blood flow distribution also increases gradually from the ventral to the dorsal side, but do not decrease significantly at last (32). In prone position, the ventilation volume of dorsal area increases, but the decrease of blood flow is not obvious, and the ratio of ventilation to blood flow is more matched. At present, it is considered that ventilation and blood flow are mainly distributed in gravity-dependent areas, ventilation and blood flow are mainly distributed in the dorsal side in supine position, and there are great differences in ventilation and blood flow in impassable position in prone position, mainly in abdomen (33). However, the difference between ventilation and blood flow in prone position is not as obvious as that in supine position, so the ratio of ventilation and blood flow is more matched after prone position. Besides, studies have shown that in the later stage of mechanical ventilation, the pulmonary shunt and physiologically ineffective cavity in the prone position are lower than those in the supine position (34, 35). These changes can make the ventilatory blood flow distribution of the lung tissue more uniform and the ventilation blood flow ratio significantly improved after the prone position (36–38).

Previous studies (39, 40) have shown that prone position ventilation can improve the ventilation of dorsal lung tissue and blood perfusion of sternal lung tissue in children with ARDS, and prone position can also play a role in draining secretions. Therefore, it is beneficial to improve the pathological state of lung tissue and promote the recovery of disease, so as to shorten the time of mechanical ventilation and improve the prognosis. In adult patients, two high-quality meta-analyses (8, 41) showed that only when the duration of continuous prone position ventilation >12 h/days, prone position ventilation could reduce the mortality of ARDS patients. For children, medical workers may consider that too long prone position may lead to other complications, such as skin damage, and the prone position they used in the study may not be as long as adults. In this study, four studies reported the effect of prone position ventilation on the mortality of children with ARDS, and two of them adopted the intervention of continuous prone position ventilation 12 h/days and 4 h/days, respectively. The results showed that prone position ventilation could reduce the mortality of children with ARDS. The analysis of the causes may be related to the different severity and prognosis of children and adults with ARDS. Therefore, the appropriate ventilation time, turning times and treatment course of children with ARDS in prone position cannot refer to the standard of adult patients, which needs to be further evaluated in the future (42, 43).

There are still some limitations to be considered in this meta-analysis. Firstly, the time span of this meta-analysis is large, and the continuous development of medical ventilation technology may affect the results. The most included authors have mentioned Berlin criteria for defining in ARDS, however, these criteria were developed for adults and published in 2012. At that time, there was no specific uniform standard for children's ARDS, leading to the difference in the diagnosis of ARDS. Secondly, there are few clinical trials related to children with ARDS, especially the lack of high-quality, multicenter randomized controlled trials. Thirdly, the sample size of this meta-analysis is relatively small, and the sample size varies greatly among studies, which may affect the reliability of synthesized results. Finally, the studies included in this meta-analysis did not grade the severity of the disease, nor did the subgroup analysis of the effects of prone position ventilation on children with different degrees of illness. The results of adult studies show that there are some differences in the therapeutic effects of prone position ventilation in different degrees of ARDS patients, so the results of this meta-analysis should be treated with cautions.

## Conclusions

In conclusion, with 7 RCTs included, this study has found that prone position ventilation is helpful to improve the arterial oxygenation pressure, PaO_2_/FiO_2,_ oxygenation index, mean airway pressure and reduce the mortality in ARDS children. Although we affirm the advantage of prone position ventilation in the improvement of oxygenation in the short term, it does not improve the duration of mechanical ventilation and long-term prognosis of children. The existing evidence does not recommend prone position ventilation as a routine mechanical ventilation method. However, its therapeutic value in severe children with acute respiratory distress syndrome is still worth looking forward to. Therefore, the exact clinical effect of prone position ventilation in children with ARDS still needs to be verified by more centers, large samples and high-quality clinical RCTs, so as to provide better evidence support for the application of prone position ventilation in children with ARDS.

## Data Availability

The original contributions presented in the study are included in the article/Supplementary Material, further inquiries can be directed to the corresponding author.

## References

[1] Khemani RG Smith L Lopez-Fernandez YM Kwok J Morzov R Klein MJ Paediatric acute respiratory distress syndrome incidence and epidemiology (PARDIE): an international, observational study. Lancet Respir Med. (2019) 7(2):115–28. 10.1016/S2213-2600(18)30344-8 30361119 PMC7045907

[2] Zheng F Pan Y Yang Y Zeng C Fang X Shu Q Novel biomarkers for acute respiratory distress syndrome: genetics, epigenetics and transcriptomics. Biomark Med. (2022) 16(3):217–31. 10.2217/bmm-2021-0749 35026957

[3] De Luca D Tingay DG van Kaam AH Courtney SE Kneyber MCJ Tissieres P Epidemiology of neonatal acute respiratory distress syndrome: prospective, multicenter, international cohort study. Pediatr Crit Care Med. (2022) 23(7):524–34. 10.1097/PCC.0000000000002961 35543390

[4] Schouten LR Veltkamp F Bos AP van Woensel JB Serpa Neto A Schultz MJ Incidence and mortality of acute respiratory distress syndrome in children: a systematic review and meta-analysis. Crit Care Med. (2016) 44(4):819–29. 10.1097/CCM.0000000000001388 26509320

[5] Smith LS Zimmerman JJ Martin TR. Mechanisms of acute respiratory distress syndrome in children and adults: a review and suggestions for future research. Pediatr Crit Care Med. (2013) 14(6):631–43. 10.1097/PCC.0b013e318291753f 23823199

[6] Prabhakaran P. Acute respiratory distress syndrome. Indian Pediatr. (2010) 47(10):861–8. 10.1007/s13312-010-0144-9 21048239

[7] Ciomartan TC. Pediatric acute respiratory distress syndrome-will we be able to predict it and eventually prevent it? Crit Care Med. (2022) 50(3):501–4. 10.1097/CCM.0000000000005324 35191869

[8] Munshi L Del Sorbo L Adhikari NKJ Hodgson CL Wunsch H Meade MO Prone position for acute respiratory distress syndrome. A systematic review and meta-analysis. Ann Am Thorac Soc. (2017) 14(Supplement_4):S280–8. 10.1513/AnnalsATS.201704-343OT 29068269

[9] Tasaka S Ohshimo S Takeuchi M Yasuda H Ichikado K Tsushima K ARDS clinical practice guideline 2021. Respir Investig. (2022) 60(4):446–95. 10.1016/j.resinv.2022.05.003 35753956

[10] Bhandari AP Nnate DA Vasanthan L Konstantinidis M Thompson J. Positioning for acute respiratory distress in hospitalised infants and children. Cochrane Database Syst Rev. (2022) 6(6):CD003645. 35661343 10.1002/14651858.CD003645.pub4 PMC9169533

[11] Lupton-Smith A Argent A Rimensberger P Frerichs I Morrow B. Prone positioning improves ventilation homogeneity in children with acute respiratory distress syndrome. Pediatr Crit Care Med. (2017) 18(5):e229–34. 10.1097/PCC.0000000000001145 28328787

[12] Page MJ McKenzie JE Bossuyt PM Boutron I Hoffmann TC Mulrow CD The PRISMA 2020 statement: an updated guideline for reporting systematic reviews. Br Med J. (2021) 372:n71. 10.1136/bmj.n71 33782057 PMC8005924

[13] Force ADT Ranieri VM Rubenfeld GD Thompson BT Ferguson ND Caldwell E Acute respiratory distress syndrome: the Berlin definition. JAMA. (2012) 307(23):2526–33. 22797452 10.1001/jama.2012.5669

[14] Shi X Sun T. Progress in diagnosis and treatment of acute respiratory distress syndrome. Chin Hosp. (2022) 9(4):4–7.

[15] Curley MA Hibberd PL Fineman LD Wypij D Shih MC Thompson JE Effect of prone positioning on clinical outcomes in children with acute lung injury: a randomized controlled trial. JAMA. (2005) 294(2):229–37. 10.1001/jama.294.2.229 16014597 PMC1237036

[16] Dong Z Lu Q Ye Z Lu H. Effect of prone position mechanical ventilation in the treatment of severe pneumonia in children. Chin Gen Pract. (2015) 13(1):69–71.

[17] Ibrahim T Elmohamady H. Prone position: how far they can improve oxygenation in pediatric patients with acute respiratory distress syndrome? J Med Sci. (2007) 7(3):390–5.

[18] Liu X Zhang Y. Clinical effect of mechanical ventilation in different positions in children with respiratory distress syndrome. J South Anhui Med Coll. (2018) 37(6):547–50.

[19] Sawhney A Kumar N Sreenivas V Gupta S Tyagi V Puliyel JM. Prone versus supine position in mechanically ventilated children: a pilot study. Med Sci Monit. (2005) 11(5):CR235–240. 15874889

[20] Sun S Xiao H Lin D Yan T Du P Tan T. Analysis of the efficacy of prone position ventilation combined with lung dilatation in the treatment of neonatal respiratory distress syndrome. Chin Mater Child Health Care. (2017) 11(12):2774–6.

[21] Wu J Zhai J Jiang H Sun Y Jin B Zhang Y Effect of change of mechanical ventilation position on the treatment of neonatal respiratory failure. Cell Biochem Biophys. (2015) 72(3):845–9. 10.1007/s12013-015-0547-2 25647746

[22] Marraro GA Chen C Piga MA Qian Y Spada C Genovese U. Acute respiratory distress syndrome in the pediatric age: an update on advanced treatment. Zhongguo Dang Dai Er Ke Za Zhi. (2014) 16(5):437–47. 24856990

[23] Kopstick AJ Rufener CR Banerji AO Hudkins MR Kirby AL Markwardt S Recognizing pediatric ARDS: provider use of the PALICC recommendations in a tertiary pediatric ICU. Respir Care. (2022) 67(8):985–94. 10.4187/respcare.09806 35728822

[24] Khemani RG Yehya N. Diagnosing pediatric ARDS still requires clinical judgment. Chest. (2023) 164(3):568–9. 10.1016/j.chest.2023.05.010 37689466

[25] Fan E Brodie D Slutsky AS. Acute respiratory distress syndrome: advances in diagnosis and treatment. JAMA. (2018) 319(7):698–710. 10.1001/jama.2017.21907 29466596

[26] Wong JJ Loh TF Testoni D Yeo JG Mok YH Lee JH. Epidemiology of pediatric acute respiratory distress syndrome in Singapore: risk factors and predictive respiratory indices for mortality. Front Pediatr. (2014) 2:78. 25121078 10.3389/fped.2014.00078 PMC4110624

[27] Bellani G Laffey JG Pham T Fan E Brochard L Esteban A Epidemiology, patterns of care, and mortality for patients with acute respiratory distress syndrome in intensive care units in 50 countries. JAMA. (2016) 315(8):788–800. 10.1001/jama.2016.0291 26903337

[28] Rowan CM Klein MJ Hsing DD Dahmer MK Spinella PC Emeriaud G Early use of adjunctive therapies for pediatric acute respiratory distress syndrome: a PARDIE study. Am J Respir Crit Care Med. (2020) 201(11):1389–97. 10.1164/rccm.201909-1807OC 32130867 PMC7258654

[29] Flores JC Imaz A Lopez-Herce J Serina C. Severe acute respiratory distress syndrome in a child with malaria: favorable response to prone positioning. Respir Care. (2004) 49(3):282–5. 14982648

[30] Yuan X Pan C Xie J Qiu H Liu L. An expanded definition of acute respiratory distress syndrome: challenging the status quo. J Intensive Med. (2023) 3(1):62–4. 10.1016/j.jointm.2022.06.002 36785583 PMC9848386

[31] Nguyen J Thompson JM Balcarcel DR Alder MN McKeone DJ Halstead ES Immunocompromised children with acute respiratory distress syndrome possess a distinct circulating inflammatory profile. Crit Care Explor. (2023) 5(1):e0844. 10.1097/CCE.0000000000000844 36699254 PMC9829269

[32] Afshin-Pour B Qiu M Hosseini Vajargah S Cheyne H Ha K Stewart M Discriminating acute respiratory distress syndrome from other forms of respiratory failure via iterative machine learning. Intell Based Med. (2023) 7:100087. 10.1016/j.ibmed.2023.100087 36624822 PMC9812471

[33] Ranieri VM Rubenfeld G Slutsky AS. Rethinking acute respiratory distress syndrome after COVID-19: if a “better” definition is the answer, what is the question? Am J Respir Crit Care Med. (2023) 207(3):255–60. 10.1164/rccm.202206-1048CP 36150099 PMC9896638

[34] Mok YH Lee JH Rehder KJ Turner DA. Adjunctive treatments in pediatric acute respiratory distress syndrome. Expert Rev Respir Med. (2014) 8(6):703–16. 10.1586/17476348.2014.948854 25119574

[35] Li X Scales DC Kavanagh BP. Unproven and expensive before proven and cheap: extracorporeal membrane oxygenation versus prone position in acute respiratory distress syndrome. Am J Respir Crit Care Med. (2018) 197(8):991–3. 10.1164/rccm.201711-2216CP 29313706

[36] Peng Q Yang S Zhang Y Zhao W Hu M Meng B Effects of awake prone position vs. usual care on acute hypoxemic respiratory failure in patients with COVID-19: a systematic review and meta-analysis of randomized controlled trials. Front Med (Lausanne). (2023) 10:1120837. 10.3389/fmed.2023.1120837 37081841 PMC10111056

[37] Katira BH Osada K Engelberts D Bastia L Damiani LF Li X Positive end-expiratory pressure, pleural pressure, and regional compliance during pronation: an experimental study. Am J Respir Crit Care Med. (2021) 203(10):1266–74. 10.1164/rccm.202007-2957OC 33406012

[38] Gleissman H Forsgren A Andersson E Lindqvist E Lipka Falck A Cronhjort M Prone positioning in mechanically ventilated patients with severe acute respiratory distress syndrome and coronavirus disease 2019. Acta Anaesthesiol Scand. (2021) 65(3):360–3. 10.1111/aas.13741 33165936 PMC7894343

[39] Shitao Z Wenjing L Chuan L. Changes of intragastric pressure in patients with severe acute respiratory distress syndrome before and after prone position ventilation. J Nurs. (2022) 34(3):11–4.

[40] Minmin Y Jianfeng Z Xiaoli G. Effect and nursing experience of early prone position ventilation in children with sepsis complicated with acute respiratory distress syndrome. Chine Gen Med. (2022) 18(11):1957–60.

[41] Guangfeng L Xueqing Z Suxia Z. Meta analysis of ventilation in prone position and supine position in patients with ALI/ARDS. J Nurs. (2016) 31(22):87–92.

[42] Coronado-Munoz A Escalante-Kanashiro R. Pediatric acute respiratory distress syndrome: how to protect the lungs during mechanical ventilation? Bol Med Hosp Infant Mex. (2021) 78(3):181–90. 34167144 10.24875/BMHIM.20000148

[43] Leroue MK Maddux AB Mourani PM. Prone positioning in children with respiratory failure because of coronavirus disease 2019. Curr Opin Pediatr. (2021) 33(3):319–24. 10.1097/MOP.0000000000001009 33782242 PMC8544610

